# Protein Intake and Frailty: A Matter of Quantity, Quality, and Timing

**DOI:** 10.3390/nu12102915

**Published:** 2020-09-23

**Authors:** Hélio J. Coelho-Junior, Emanuele Marzetti, Anna Picca, Matteo Cesari, Marco C. Uchida, Riccardo Calvani

**Affiliations:** 1 Università Cattolica del Sacro Cuore, 00168 Rome, Italy; 2 Applied Kinesiology Laboratory-LCA, School of Physical Education, University of Campinas, 083-851 Campinas-SP, Brazil; uchida@unicamp.br; 3 Mãe Mariana Nursing Home, Rehabilitation Unit, 08562-460 Poá-SP, Brazil; 4 Fondazione Policlinico Universitario “Agostino Gemelli” IRCCS, 00168 Rome, Italy; anna.picca@guest.policlinicogemelli.it (A.P.); riccardo.calvani@guest.policlinicogemelli.it (R.C.); 5 Department of Clinical Sciences and Community Health, Università di Milano, 20133 Milan, Italy; macesari@gmail.com; 6 Geriatric Unit, Fondazione IRCCS Ca’ Granda Ospedale Maggiore Policlinico, 20122 Milan, Italy

**Keywords:** aging, diet, physical function, disability, sarcopenia, nutrition, amino acids, metabolism, dietary patterns, protein per meal

## Abstract

Frailty is a geriatric syndrome that refers to a state of reduced resiliency to stressful events that occurs in response to physiological and/or psychosocial detriments. Frailty is a predictor of poor prognosis, given that frail older adults are at higher risk of many adverse health-related events. Hence, the identification of potential strategies to prevent the development and progression of frailty is of extreme importance for avoiding its negative outcomes. An adequate protein consumption is advocated as a possible intervention for the management of frailty in older adults due to its effects on muscle mass and physical function. However, empirical evidence is still needed to support this proposition. On the other hand, substantial evidence from observational studies has provided important information on the association between frailty and dietary protein-related parameters. Here, we provide a narrative review of the current literature regarding the association between protein intake (amount (how much?), quality (what type?), and distribution across meals (when?)) and frailty-related parameters. The ultimate aim of this work is to offer practical, evidence-based indications to healthcare professionals responsible for the care of frail older adults.

## 1. Introduction

Frailty refers to a state of reduced resiliency to stressful events that occur as a consequence of multisystem derangements and poor social support [1,2,3,4]. This condition is highly prevalent in older adults, especially among hospitalized and institutionalized people, in particular in low- and middle-income regions [5]. As frailty progresses, people become increasingly more vulnerable to numerous adverse health-related events, including falls and fractures, cognitive decline, disability, hospitalization, nursing home placement, and death [6,7,8,9,10,11,12]. Hence, the identification of potential strategies to prevent the development and progression of frailty is of the utmost importance for avoiding its negative outcomes.

A high dietary protein intake is recognized as a possible intervention for the management of frailty in older adults due to its effects on muscle mass and physical function [2,13]. Yet, randomized clinical trials in support of high protein ingestion are scanty [2,14]. On the other hand, substantial evidence from observational studies has provided important information on the association between frailty and dietary protein-related parameters.

Here, we provide an overview of the current literature regarding the association between dietary protein intake and frailty in older adults. In particular, we describe the influence of the amount (how much?), quality (what type?), and distribution of dietary protein across meals (when?) on frailty and frailty-related parameters. The ultimate aim of this work is to provide practical, evidence-based indications to healthcare professionals responsible for the care of frail older adults.

## 2. Proteins

Protein composition, absorption, and degradation are a large topic that has been a field of research for many scientists around the world. This subject is predominately discussed in classes for nutritionists and endocrinologists and, most of the times, poorly debated in the training of other health professionals. However, such knowledge has become essential due to the impact of protein intake on health-related parameters in older adults. Hence, a basic knowledge of the processes associated with protein metabolism and amino acids (AAs) availability is necessary to understand the main topics of the present review and discuss them in an interdisciplinary team. Hence, a short introduction to this theme is provided in the following paragraphs.

Proteins are macromolecules with a pivotal role in many physiological processes involved in body homeostasis, including structure, function, synthesis, restoration, and transportation. Although the other macronutrients—carbohydrates and fat—may be stored in the body to be used in situations of need, protein cannot be deposited into an inactive compound (e.g., glycogen) to serve as a reservoir. Consequently, dietary protein consumption must be equivalent to bodily metabolic demands to prevent the use of skeletal muscle contractile proteins as sources of AAs in situations of stress and fasting. Hence, an adequate supply of dietary proteins is crucial to maintain body homeostasis and function.

**Figure 1:**
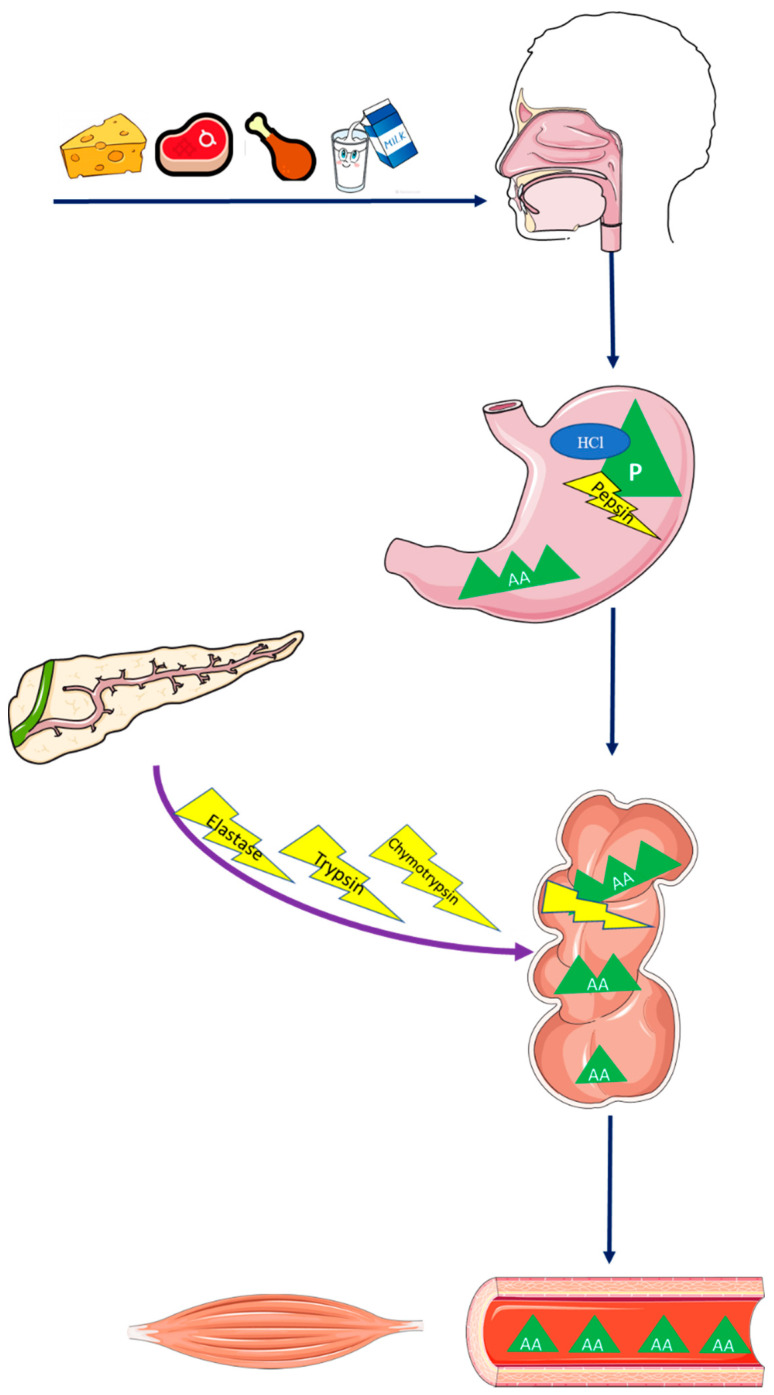
Protein absorption and digestion. Abbreviations: AA, amino acid; HCl, hydrochloric acid; P, protein.

Indeed, essential AAs (EAAs) are not produced by the human body and must be acquired through their extraction from dietary protein (e.g., eggs, milk, cheese). The degradation of protein into small molecules also differs from the metabolism of other macronutrients by beginning in the stomach. This process is called proteolysis and has the direct participation of hydrochloric acid (HCl), which contributes to the proteolytic action of digestive enzymes by revealing peptide bonds. These peptide bonds are cleaved into smaller AA molecule chains by pepsin, an enzyme activated by the action of HCl. In the duodenum, trypsin, chymotrypsin, elastase, and carboxypolipeptidase, enzymes produced in the exocrine part of the pancreas, support the degradation process by cleaving AA chains into tripeptides and dipeptides. Although tripeptides and dipeptides are smaller than the AA chains found in the stomach, they still need to be converted into a small and simple molecule of AA in order to then be absorbed into the bloodstream and transported to target tissues. A schematic representation of protein absorption and digestion is depicted in Figure 1.

## 3. Age-Related Muscle Atrophy, Sarcopenia, and Frailty

The skeletal muscle is the largest organ of the human body and constitutes almost 50% of the total body mass. The muscle is not only the mechanical apparatus of locomotion but represents the largest protein “emergency” reservoir in the body and has a pivotal role in the regulation of energy metabolism [15]. Recently, its importance as an active endocrine organ that synthesizes and releases numerous molecules, collectively called myokines, has been widely acknowledged [16].

Muscle mass is regulated by the dynamic and transient equilibrium between muscle protein synthesis (MPS) and muscle protein breakdown (MPB) so that it remains virtually unvaried when the net balance is zero. Food intake, mainly protein ingestion, is a major regulator of muscle protein metabolism [13,17,18,19,20]. The increased AA availability stimulates myofibrillar and sarcoplasmic MPS [13,17,18,19,20] through the activation of ribosomal protein kinase S6 (S6K1) and 4E-binding protein 1 (4EBP1), under the coordination of the mammalian target of rapamycin (mTOR) [17,20,21].

**Figure 2:**
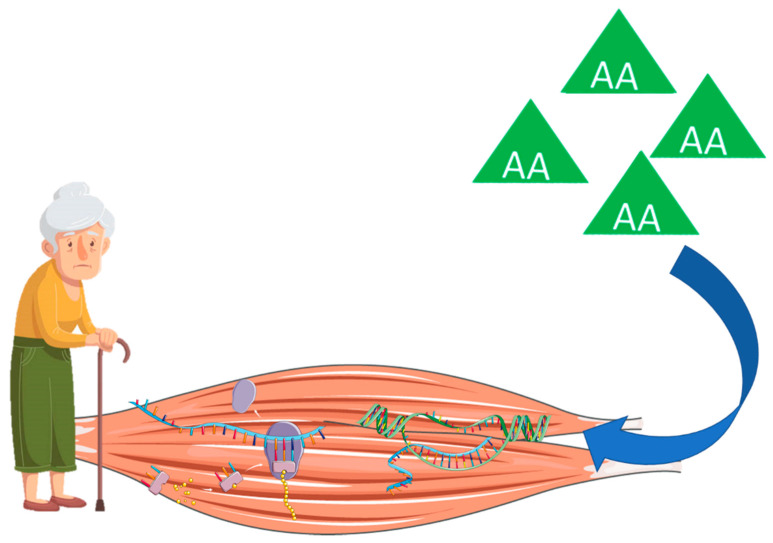
Anabolic resistance to protein intake in older adults. Abbreviation: AA, amino acid.

Notably, the old muscle requires greater amounts of AAs to stimulate muscle anabolism due to a reduced MPS in response to hyperaminoacidemia [22,23,24,25], a phenomenon known as anabolic resistance (Figure 2). According to Moore et al. [26], up to 140% higher protein intake might be required by older adults in comparison to young people to maximally stimulate postprandial rates of MPS. Such a reduced capacity of the old muscle to trigger protein synthesis is reflected by a markedly lower activation of anabolic signaling pathways, such as mTOR and S6K1, after infusion of EAAs [27]. If the anabolic resistance is not overcome by a proportionally higher protein intake, reductions in MPS are expected, causing an imbalance in muscle metabolism that favors MPB and atrophy [28].

Age-related muscle wasting has important clinical implications due to its close relationship with declining physical function. Although impaired physical performance in older adults is not fully explained by muscle atrophy [29], the loss of muscle mass during aging preferably affects type II fibers [30,31,32]. These fibers have greater content and higher activity of myosin ATPase and glycolytic enzymes in comparison to type I muscle fibers [33], which allows them to produce maximal strength and power and influence mobility [34,35].

Muscle atrophy is also a cardinal element in the development of sarcopenia, also called muscle failure [36], a degenerative neuromuscular disease that involves significant muscle atrophy, loss of muscle strength, and physical dysfunction [37]. Sarcopenia is recognized as a major public health problem, given its association with an increased risk for disability, institutionalization, and death [38,39], with high prevalence in older adults [40,41,42] and in people with premature aging [43]. Moreover, sarcopenia is associated with high healthcare costs (e.g., hospitalization, nursing home admissions), representing $18.5 billion for the United States Government in 2000 [44].

The progression of sarcopenia may also open the door to the development of other conditions, such as frailty [45]. Frailty is a geriatric syndrome characterized by a reduced capacity of the human body to cope with stressful conditions, which occurs in response to a nonlinear multisystem physiological dysregulation and poor psychosocial support [1,2,3,4]. Frailty might occur in adults but is highly prevalent in the older population [5]. As frailty evolves, people become more vulnerable to many negative outcomes [1,2,3,4]. Although the theoretical construct of frailty and its clinical importance have been well established, its operationalization is still hampered by the absence of a univocal definition. In fact, many instruments for the identification of frailty are available, with limited concordance across them [46,47].

The phenotypic model proposed by Fried et al. [48] is the most used operational definition of frailty in research and clinical practice [3,4]. This model was developed by examining more than 5000 participants from the Cardiovascular Health Study (CHS). The frailty phenotype was created based on five cardinal features: (a) unintentional weight loss; (b) muscle weakness; (c) self-reported fatigue; (d) impaired mobility, and (e) sedentary behavior. Researchers established that people with three or more factors should be identified as frail, while those with one or two features should be considered pre-frail. Researchers also observed that being frail according to their classification was associated with an increased risk of physical dysfunction, disability, hospitalization, and death [48]. These findings were expanded by numerous studies [6,7,11,49,50,51,52], such that it is currently recognized that frailty is also associated with cardiovascular abnormalities, depressive symptoms, cognitive dysfunction, fractures, and nursing home placement.

**Figure 3:**
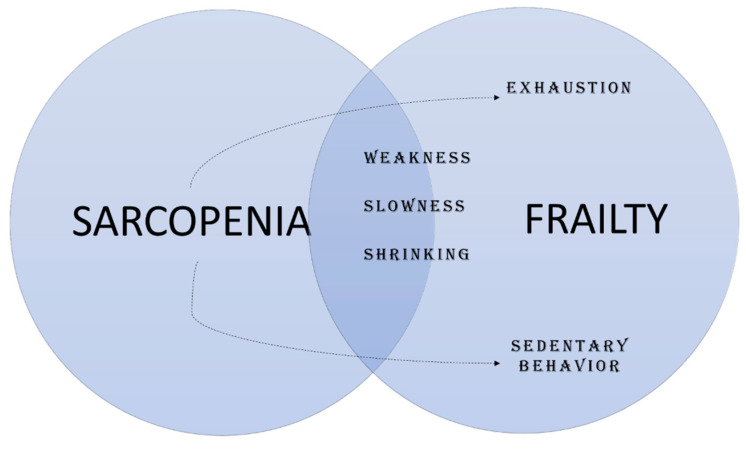
Sarcopenia and frailty.

Notably, sarcopenia and frailty share many clinical features [45], including loss of muscle strength, physical dysfunction, and body shrinking [53]. Besides these, exhaustion and reductions in physical activity levels commonly occur with the progression of sarcopenia (Figure 3). Hence, experts in the field have suggested that sarcopenia might be seen as a precursor for the development of frailty [45]. In other words, frailty may be the product of sarcopenia progression [45]. This idea is further supported by the higher prevalence of sarcopenia in pre-frail and frail older adults when compared with robust people [54,55].

## 4. How Much? Current Recommendations and Available Evidence

The Estimated Average Requirement (EAR) and the Recommended Dietary Allowances (RDA) are two reference parameters proposed by the National Academies of Sciences, Engineering, and Medicine (NASEM) that can be used for planning and assessing diets [56]. EAR refers to the average daily intake that is sufficient to meet the nutrient requirement of half of the healthy individuals, while the RDA is thought to be sufficient to meet the nutrient requirement of almost all healthy individuals according to age and gender. The current EAR and RDA for protein intake are 0.6 g/kg body weight (BW)/day and 0.8 g/kg BW/day, respectively.

However, the value of RDA is debated [13,57,58,59,60,61] since its establishment was based on nitrogen balance studies. Besides, RDA does not offer specific recommendations for older adults, who seem to need higher intakes of protein to maintain metabolic homeostasis. The nitrogen balance method is based on the fact that proteins are fundamentally composed of nitrogen, which, after being metabolized through transamination and deamination reactions, is mainly excreted in the urine and, in smaller quantities, in the feces and skin [56]. According to this paradigm, when nitrogen intake exceeds its losses, a positive balance is achieved, which favors MPS [56]. On the other hand, a greater excretion of nitrogen defines a condition of negative nitrogen balance, which is assumed as a catabolic state [56].

The main criticisms to this method are related to (a) slow rate of urea turnover in adults, which requires several days to adapt to changing levels of protein intake; (b) the apparent retention of nitrogen by adults, increasing the risk of type I error; (c) the need to include dermal losses of nitrogen in the final calculation; (d) the accuracy and attention required to perform the evaluation [56].

Besides, empirical evidence using short-term balance approaches (5–10 days) indicates that diets based on the current RDA for protein intake are insufficient to maintain the nitrogen balance [62,63]. Gersovitz et al. [63] observed that men and women who consumed 0.8 g/kg BW/day of egg protein were in negative nitrogen balance. Campbell et al. [62] expanded this finding by indicating that at least 1.0 g/kg BW/day of protein would be necessary to match protein requirements for positive nitrogen balance.

Taken together, these findings suggest that the current protein RDA might be insufficient to prevent muscle atrophy in older adults, contributing to the development of sarcopenia and frailty. Hence, opinion articles and consensus statements have argued that of a dietary protein greater than the RDA (1.0–1.5 g/kg BW/day) should be recommended to prevent or postpone age-related muscle atrophy [64,65,66] and neuromuscular decline [57,58,59,60,61,67]. These premises are based on several studies in which older adults who reported protein intake higher than the RDA showed better physical function [68,69,70,71,72,73].

Such associations between protein intake and sarcopenia-related parameters led researchers to propose that a high protein diet could also affect frailty status in older adults. Many observational have confirmed this hypothesis by reporting an inverse association between protein consumption and frailty status in older adults.

Nanri et al. [74] reported that protein intake was inversely associated with frailty prevalence in Japanese men and women. Rahi et al. [75] found that high protein intake (≥1.0 g/kg BW/day) was associated with 59% lower frailty prevalence, after adjustment for covariates, in French old community-dwellers. Sandoval-Insausti et al. [76] analyzed data of the Seniors-ENRICA (Study on Nutrition and Cardiovascular Risk Factors in Spain) cohort study in community-dwelling older adults (≥60 years) and reported increasing odds ratios of frailty for reduced protein intake levels. The authors observed that the prevalence of frailty was lower in older adults with an average protein intake of 1.28 g/kg BW/day. Beasley et al. [77] provided longitudinal evidence by investigating 24,417 women of the Women’s Health Initiative Observational Study (WHI-OS). Researchers reported that a protein consumption of 1.2 g/kg BW/day was associated with a lower risk of incident frailty over three years of follow-up. These findings are supported by a systematic review and meta-analysis that included more than 18,000 community-dwelling older adults from five different countries [78].

Although these findings support the need for increasing protein intake in older adults to avoid frailty, data should be carefully interpreted in light of some important considerations [78]. The main limitation is that the relationship between protein intake and frailty is not unanimous among studies [74,79,80], and some authors [79] have suggested that protein distribution over the day and/or protein quality, an index of the amount of EAAs that is provided by a given quantity of protein [81], may be more relevant to muscle anabolism than overall protein intake. Moreover, these studies used different instruments for assessing frailty, which might indicate that they captured different frailty domains [82].

## 5. What Type? Animal-Based vs. Plant-Based Protein

**Figure 4:**
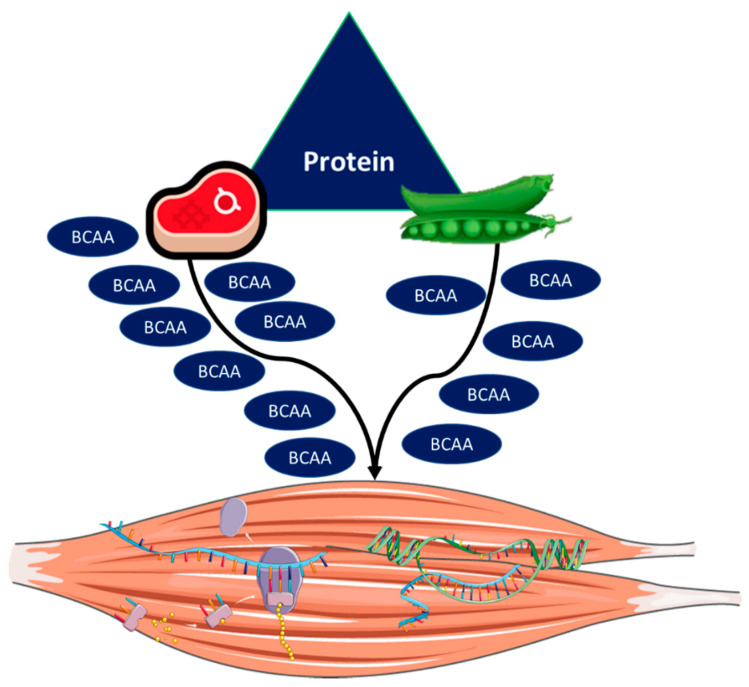
Animal- and plant-based protein sources. Animal-based plant protein is expected to have a higher content of branched-chain amino acids (BCAAs), thereby evoking greater stimulation of anabolic pathways and muscle protein synthesis than plant-based protein.

Protein quality refers to the anabolic response induced by a specific protein source, which can be of animal (e.g., milk, eggs, meat) or vegetal (e.g., soy, wheat) origin [83]. Protein quality has become a trending topic in the field of nutrition and aging, given that plant-based diets (e.g., vegetarian) have gained considerable popularity [84] and researchers have argued that this type of diet may be more sustainable by causing lower environmental impact than animal-based diets [83,85]. However, a major concern with this approach resides in the fact that plant-based protein elicits lower MPS in comparison to protein from animal sources [19] (Figure 4).

Anthony et al. [86] reported that whey protein (WP) caused significantly higher phosphorylation of molecules with a key role in MPS, including mTOR, eIFE, 4E-BP1, and S6K1, than soy protein (SP) in rats. Mitchell et al. [87] confirmed and expanded these findings by indicating that WP elicited longer phosphorylation of the ribosomal protein S6K1 in comparison to SP in humans. Moreover, Tang et al. [19] observed that acute intake of a WP hydrolysate induced greater MPS than soy consumption in healthy adults. In older people, Yang et al. [88] reported that neither 20 g nor 40 g of SP stimulated MPS under resting conditions, while myofibrillar MPS was significantly increased after ingestion of the same doses of WP. Researchers also reported greater MPS after resistance exercise combined with WP compared with SP plus physical exercise.

The different anabolic responses elicited by animal- and plant-based protein may be attributed to (a) digestion and absorption kinetics and (b) EAA content, mainly branched-chain amino acids (BCAAs; i.e., isoleucine, leucine, and valine). Digestibility rates, i.e., the proportion of dietary protein-derived AAs that is effectively digested and absorbed, becoming available in a form suitable for body protein synthesis, are significantly different between animal and plant protein sources [83,85]. Indeed, digestibility rates higher than 90% are expected with animal-based protein [83,85]. On the other hand, less than 50% of digestibility is commonly observed with plant-based protein [83,85].

A possible explanation for this observation may be the fact that plant proteins, mainly soy, are rapidly digested, causing a marked increase in the transportation of AAs to the liver [89]. Most of this AA pool undergoes transamination and oxidative deamination reactions to produce ammonia and urea, dramatically reducing AA availability for MPS [89]. Unfortunately, only a few studies have investigated AA kinetics according to protein sources due to the high cost associated with the use of intrinsically labeled protein sources and further studies are needed in order to better delineate this phenomenon [90].

Animal-derived protein (e.g., meat, eggs) is believed to have a higher content of EAAs and BCAAs, thereby evoking greater MPS than plant-based protein (e.g., soya, beans, nuts) [13,83]. Hence, animal foods are recognized as the primary source of high-quality protein [64,91,92]. EAAs play a key role in the anabolic response elicited by feeding [93,94], given that the administration of non-EAAs does not stimulate MPS, whereas muscle anabolism rates near to 90% are observed after EAA infusion [94]. Among EAAs, much attention has been paid to BCAA intake, mainly leucine (reviewed in [95]).

Leucine is considered to be a major stimulator of muscle anabolism [21,96,97]. Acute oral administration of leucine increased protein synthesis in a dose-dependent way in myoblasts [96] and in the rat muscle [96,97]. Similar findings have been reported in humans. Rieu et al. [98] observed that leucine supplementation acutely increased MPS in older adults. Moreover, Atherton et al. [99] found that leucine administration elicited greater muscle anabolic responses after exercise in young and old adults compared with participants supplemented with alanine.

The effects of leucine on muscle plasticity are mediated by its actions on the main anabolic pathways regulating protein synthesis in skeletal muscle [96,97,100]. In fact, leucine stimulates mTOR [100] and p70 [96,100]. Downstream proteins of the mTOR pathway, including 4E-BP1, eIF4E, and S6K1, are also phosphorylated in response to leucine bioavailability [96,97,100]. On the other hand, these anabolic effects were abolished by rapamycin, an inhibitor of mTOR [100].

Although there is no consensus on which of the two factors (digestion and absorption kinetics or EAA content) is more relevant to muscle anabolism, Wilkinson et al. [101] provided interesting results for this discussion by comparing the effects of nonfat milk and isonitrogenous SP with similar amounts of EAAs. Based on their findings, the authors concluded that milk protein promoted greater and more sustained MPS than soy, suggesting a key role for protein digestion rates in subsequent anabolic responses [101]

Results from epidemiological studies on the relationship between animal versus plant protein intake and sarcopenia-related parameters are inconsistent. Muscle mass has been investigated as the primary outcome of interest in many studies. Animal and plant protein intakes were significantly associated with muscle mass in middle-aged and older adults [91]. A case-control study found greater muscle mass in omnivores compared with vegetarian women [102]. Further analyses indicated that animal, but not vegetal protein intake was significantly associated with muscle mass index, regardless of dietary patterns [102]. Similarly, Gingrich et al. [66] observed that women with low skeletal muscle index (SMI) consumed more plant protein and less animal protein than those with normal SMI. Finally, Alexandrov et al. [103] found a significant association between animal protein consumption and muscle mass in 31,278 men and 45,355 women from the Lifelines Cohort.

Few longitudinal studies have assessed the association between protein quality and changes in muscle mass over time. Findings from the Health, Aging, and Body Composition (Health ABC) study [65] indicated that absolute and animal protein intake were significantly associated with changes in muscle mass in community-dwelling adults, while no significant associations were observed with vegetal protein. However, Chan et al. [64] reported that total and animal protein intakes were not associated with changes in physical performance and appendicular skeletal muscle (ASM) over a 4-year follow-up in Chinese community-dwelling older adults. However, people who had a relative vegetal protein consumption higher than 0.72 g/kg BW/day lost significantly less ASM during the follow-up.

Regarding physical performance, Coelho-Junior et al. [92] reported that fast-paced walking speed was inversely associated with relative animal protein consumption and positively correlated with the intake of plant-based protein in Brazilian older adults. In contrast, a higher dietary intake of animal protein was associated with reduced loss of handgrip strength over six years in a cohort of the Framingham study [104].

Discrepancies among studies may reflect different designs, population characteristics (e.g., age, setting, ethnicity), inclusion of covariates (e.g., physical activity levels), methods for muscle mass assessment (e.g., bioimpedance, dual-energy X-ray absorptiometry [DEXA]), physical function parameters considered, and diet assessment methods (e.g., food frequency questionnaires, 24-h recall).

A question that remains unanswered is whether protein sources are associated with frailty in older adults. Unfortunately, this topic has only recently gained interest. The associations between animal protein and sarcopenia-related parameters support the idea that this protein source should be prioritized in older adults. Results from the Seniors-ENRICA study [76] are in line with this perspective by demonstrating that higher animal protein intake reduced the risk of frailty in community-dwelling older adults. However, findings from the Health ABC study suggested that a 10-g lower vegetable protein intake was associated with a 20% higher incidence of pre-frailty or frailty over four years, while no significant associations were observed with animal protein [105]. Furthermore, animal protein was only associated with incident physical inactivity, while the occurrence of slowness, weight loss, and physical inactivity was significantly associated with vegetal protein.

A possible explanation for these findings may reside in the fact that participants of the Health ABC study showed a higher intake of vegetal protein than those of the ENRICA study, suggesting that the co-ingestion of protein from foods such as soy, bean, and nuts could provide the quantity of EAAs, BCAAs, and leucine requested for stimulating muscle anabolism, reducing the risk of sarcopenia and, thus, frailty [92,105].

An important aspect to be considered in practice is that several factors may impact the consumption of animal-based protein in older adults, such as oral health, price, and even lifestyle. Cultural and regional values are also associated with dietary patterns [106,107,108] and may negatively influence the adherence to dietary recommendations [109] as well as health-related outcomes [110]. In fact, our group [111] recently compared dietary habits between Brazilian and Italian older women and found a higher vegetal protein intake in the former, while animal protein consumption was higher in Italians.

Meals rich in legumes and vegetables are commonly consumed by Brazilians [112,113] and can explain the higher intake of plant-based protein observed in this population. Notably, this pattern of diet is also highly consumed in many Asian (e.g., Thailand, Korea), African (e.g., Zimbabwe, Namibia), and South American countries (e.g., Paraguay, Chile) [114]. These dietary habits may be linked to the great symbolic value attributed to meat in low-income countries due to the remnants of monarchy and slavery [115]. In wealthy countries, the preference toward plant and animal protein might change according to the geographic region [106]. In Southern Italy, for example, people are more adherent to the Mediterranean diet, characterized by high consumption of vegetables, fruit, legumes, nuts, and unprocessed cereals and moderate consumption of fish, eggs, and wine, than people from the north [106].

Overall, these findings indicate that protein recommendations for counteracting frailty should consider the importance and impact of cultural values to foster adherence to healthier eating habits.

## 6. When? Protein Distribution across Meals

Older adults are required to consume larger amounts of protein to maintain muscle anabolism and avoid negative health-related outcomes. However, achieving such requirements is a major challenge for frail older people, also given the high prevalence of oral health problems (e.g., chewing and swallowing difficulties) observed in this population [79].

A possible approach to meet such requirements could be to provide a high protein meal, pulse-feeding, which is thought to saturate the splanchnic sequestration leading to higher availability of AAs for MPS [73]. However, studies found that MPS reached a plateau at ~30 g of protein intake per meal [116,117], after which there was a marked stimulation of whole-body AA oxidation [116]. According to Symons et al. [117], meals providing large amounts of protein (e.g., 90 g) have no greater effects on MPS than moderate meal servings (e.g., ~30 g of protein).

Hence, researchers suggested that a spread feeding pattern with at least 30 g of dietary protein during the main meals (i.e., breakfast, lunch, and dinner) could be a more effective strategy to counteract age-related muscle atrophy and strength loss in older adults than a pulse-feeding approach, besides being a more feasible method to provide the amount of recommended protein.

Ten Haaf et al. [73] observed that total protein intake was not associated with either handgrip strength or Short-Physical Performance Battery (SPPB) scores, while a more spread protein distribution in the main meals (19–26 g per meal) was related to faster WS in community-dwelling older adults. These findings were expanded by Loenneke et al. [118] who reported that consuming two or more meals with 30 g of protein each was associated with greater muscle strength and muscle mass in older adults compared with the consumption of one or no meal with at least 30 g of protein. Along similar lines, the consumption of two or three meals per day with adequate protein content (≥30 g) was associated with a lower risk of physical disability in Mexican older adults without functional limitations [119]. Findings from longitudinal studies are controversial, given that a spread protein intake was associated with greater muscle mass and strength but not better mobility over two years of follow-up [120,121].

Only one study investigated frail older adults. Bollwein et al. [79] did not observe a significant association between frailty status and absolute protein intake in community-dwelling older adults. However, the authors reported that pulse-feeding protein intake, with lower protein consumption at breakfast and higher intake at lunch, was more frequently observed in pre-frail and frail people.

## 7. Future Perspectives

The knowledge of the relationship between protein intake and frailty is growing constantly and substantial evidence has accumulated in the last few years. However, there is still a need for more information beyond the simplistic view of the amount of required protein. In fact, only a few cross-sectional studies investigated the association between protein quality and distribution across meals with frailty prevalence, limiting inferences to clinical practice. In addition, the absence of follow-up studies does not allow for conclusions to be drawn regarding possible cause-effect relationships.

Another important limitation is the lack of studies that included the assessment of several dietary protein-related parameters in the same investigation, which could provide a more comprehensive appraisal of this relationship and, consequently, the generation of more specific guidelines. Similarly, few studies have taken into consideration the influence of sociodemographic variables on the quality of dietary protein. Future reviews are required to assess the current state of the art about ethnic-specific disparities in protein consumption and which variables still need to be better investigated. This information might have a key role to foster the adherence to nutritional recommendations.

Notably, a recent systematic review and meta-analysis indicated that different instruments had been used for assessing frailty [82]. This topic deserves attention, given that negative health-related events (e.g., disability and hospitalization) are differentially predicted by various frailty instruments. A recent study by our group [122] tried to overcome these limitations by investigating the associations between frailty status according to three different frailty assessment tools and (a) daily protein intake, (b) daily BW-adjusted protein intake, (c) BCAA consumption, (d) evenness of protein distribution across main meals, and (e) number of daily meals providing at least 30 g of protein in community-dwelling older adults. The assessment tools used were the Fried’s frailty phenotype, the FRAIL scale, and the Study of Osteoporotic Fracture (SOF) index, which differ in the method used to assess physical function. We observed a frailty instrument-dependent relationship between frailty status and protein-related dietary parameters, reflected by the fact that protein consumption and BCAA intake were associated with frailty status only in participants identified as frail according to the Fried’s frailty phenotype.

Hence, future studies should focus on the simultaneous assessment of several dietary protein-related parameters, rather than simply looking at the amount of protein. Studies should also identify frailty using different instruments (e.g., Fried’s frailty phenotype, SOF, FRAIL) and phenotypes (e.g., physical and multidimensional).

Although studies have suggested that a high protein intake might be associated with a low frailty prevalence, extrapolations should be made carefully, given that specific recommendations are necessary according to the presence of comorbidities. In people with chronic kidney disease (CKD), for example, very low (~0.4–6 g/kg BW/day) and low (~0.6–8 g/kg BW/day) protein diets have been associated with a lower incidence of end-stage renal disease and mortality [123,124], while a high dietary protein intake (≥8 g/kg/d) is believed to induce glomerular injury [125]. Particularly, abnormalities in kidney function have been observed in frail older women [126], suggesting that a holistic clinical evaluation should be conducted before recommending changes in protein intake.

The consumption of other macronutrients should also be taken into consideration when planning changes in protein intake. Indeed, studies have indicated that increased energy intake mediates the relationship between protein intake and frailty [127,128,129]. According to findings from observational studies [130], older adults under long-term low energy intake are more likely to be frail, regardless of macronutrient consumption. Besides, researchers [130] have reported that the association between the intake of macronutrients and frailty-related parameters was no longer significant after adjusting for energy intake, indicating that energy has a key role in this relationship.

An adequate energy intake is normally reached through the consumption of fat and carbohydrate. During aging, however, a decline in oral health and the loss of appetite might promote the development of malnutrition and underweight [127,128,131]. Both underweight and malnutrition are among the criteria for the diagnosis of frailty [48,132], besides being associated with other frailty-related parameters, such as physical function impairment and weakness [133], likely promoting a sedentary behavior [134]. Moreover, a negative energy balance contributes to muscle atrophy and bone loss, providing a suitable environment for the development of sarcopenia and osteoporosis, respectively, two diseases closely associated with frailty development and progression [128].

Veganism, covering a variety of diet patterns that involve the avoidance of meat, has gained increasing popularity and represents a growing social movement [135]. Adherence to a vegan diet, even if only for short periods (7 weeks), might beneficially affect several cardiovascular risk factors [136,137], including BW, body mass index, systolic and diastolic blood pressure, total and high-density cholesterol, and C-reactive protein, as well as ameliorate the quality of life [137]. Of particular interest are findings that indicate similar exercise capacity among vegan, lacto-ovo-vegetarian, and omnivorous recreational runners, suggesting that an optimal physical performance might be reached with different diet patterns. These health benefits may be valuable for older adults, including frail people, and deserve urgent investigation.

The implementation of innovative, minimally invasive technologies (e.g., novel stable isotope tracer combined with “virtual biopsy” and deuterated-creatine dilution methods) will help address issues related to skeletal muscle protein kinetics and muscle mass assessment [138,139]. This will be instrumental for evaluating the efficacy of nutritional intervention designed to counteract sarcopenia and frailty [140].

Lastly, heterogeneity in response to specific nutrients and dietary patterns among older adults may exist [141,142]. The implementation of integrated “omics” approaches may lead to more accurate dietary assessments [143] and the elaboration and monitoring of personalized dietary plans, tailored to an individual’s metabolic phenotypes and needs [144]. This information could represent a critical step to guide future scientific consensus-based, personalized dietary counseling [142].

## 8. Conclusions

Protein-related parameters are associated with frailty in older adults. Many studies have described an inverse association between the amount of protein intake and frailty prevalence, leading experts in the field to suggest that greater amounts of protein than the current RDA (1.0–1.5 g/kg BW/day) are necessary to prevent frailty. Protein source may also impact the development and progression of frailty, with animal-based protein believed to provide a higher amount of BCAAs than plant-based protein, consequently evoking greater anabolic responses. However, plant protein is preferably consumed in some countries and studies have supported its positive association with physical function. Hence, cultural values should be taken into consideration in future guidelines to increase adherence. Few studies have explored the association between protein distribution across meals and frailty. Most of them found that the consumption of at least 30 g of protein in two or more meals might be more effective at preserving muscle mass and physical performance compared with the consumption of a single high-protein meal. However, the only study of such a kind conducted in frail older adults provided different results. The optimal distribution of protein across meals is an important topic to be addressed for the achievement of protein requirements in older adults. Finally, future studies should use a longitudinal design, combine the assessment of several protein-related parameters, instead of only the amount of protein intake, and identify frailty using different instruments.
